# An overview of different methods to establish a murine premature ovarian failure model

**DOI:** 10.1002/ame2.12477

**Published:** 2024-09-01

**Authors:** Negar Pouladvand, Mahnaz Azarnia, Hadis Zeinali, Rouhollah Fathi, Somayeh Tavana

**Affiliations:** ^1^ Department of Embryology, Reproductive Biomedicine Research Center Royan Institute for Reproductive Biomedicine, ACECR Tehran Iran; ^2^ Department of Animal Biology, Faculty of Biological Sciences Kharazmi University Tehran Iran

**Keywords:** animal model, chemotherapy drugs, mouse, ovarian damage, ovarian reserve, premature ovarian failure

## Abstract

Premature ovarian failure (POF)is defined as the loss of normal ovarian function before the age of 40 and is characterized by increased gonadotropin levels and decreased estradiol levels and ovarian reserve, often leading to infertility. The incomplete understanding of the pathogenesis of POF is a major impediment to the development of effective treatments for this disease, so the use of animal models is a promising option for investigating and identifying the molecular mechanisms involved in POF patients and developing therapeutic agents. As mice and rats are the most commonly used models in animal research, this review article considers studies that used murine POF models. In this review based on the most recent studies, first, we introduce 10 different methods for inducing murine POF models, then we demonstrate the advantages and disadvantages of each one, and finally, we suggest the most practical method for inducing a POF model in these animals. This may help researchers find the method of creating a POF model that is most appropriate for their type of study and suits the purpose of their research.

## Graphical abstract

Ten different methods to induce murine POF models: (1) POF model induced by chemotherapy drugs, (2) radiotherapy‐induced POF model, (3) genetically induced POF model, (4) POF model induced by D‐galactose, (5) natural ovarian aging (NOA)‐induced POF model (6) physiological POF model with consecutive superovulation, (7) oophorectomy POF model, (8) POF model induced by 4‐vinylcyclohexene diepoxide (VCD), (9) autoimmunity induced POF model, (10) psychological stress POF model.

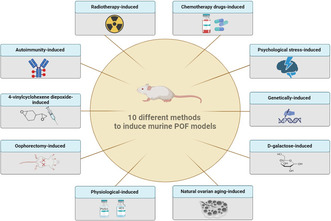

## 1 INTRODUCTION

Premature ovarian failure (POF) is a common endocrine disease that causes female infertility. The patient goes through stages such as premature ovarian insufficiency (POI) and then, with further progression of the disease, eventually develops premature and complete ovarian failure or POF.^1^ POF is characterized by high gonadotropin expression (follicle‐stimulating hormone (FSH) ≥ 40 IU/mL), low estradiol (E2) expression, follicular dysplasia, and amenorrhea for at least four months in women younger than 40 years.^2^, ^3^ In addition, patients suffer from long‐term consequences of hypoestrogenism, including osteoporosis, cardiovascular diseases, and mental disorders such as depression.^4^, ^5^ The causes of POF are unknown and are related to many complex factors such as genetic defects, autoimmune disorders, infection, iatrogenic factors (ovarian surgery, chemotherapy, or radiotherapy injury), etc.^6^, ^7^ The phenotype of POF is significantly dependent on hormonal changes. Low levels of sex hormones (estrogens and inhibins) and increased levels of gonadotropins (luteinizing hormone (LH) and FSH) (hypergonadotropic amenorrhea) are well documented as causes of POF.^8^, ^9^

As mentioned above, one of the causes of POF is iatrogenesis, which occurs during cancer chemotherapy and radiotherapy. Chemotherapy agents have become one of the important causes of premature ovarian failure in patients who present with cessation of ovarian function.^10^ Chemotherapy drugs are divided into five groups: (1) alkylating agents with a high risk of infertility (such as cyclophosphamide, mechlorethamine, chlorambucil, procarbazine, busulfan and melphalan); (2) platinum‐based compounds with an average risk of infertility (such as cisplatin and carboplatin); (3) antimetabolites with a low risk of infertility (such as methotrexate, 5‐fluorouracil, and cytarabine); (4) vinca alkaloids with a low risk of infertility (such as vincristine and vinblastine); (5) anthracycline antibiotics with a low risk of infertility (such as daunorubicin, bleomycin, and doxorubicin (moderate risk of infertility)).^11^, ^12^ Chemotherapy agents can cause double‐strand breaks (DSBs) in DNA. If a DBS is successfully repaired, the cell survives through loss of ataxia‐telangiectasia mutated (ATM) kinase. However, if repair pathways fail, DNA damage can lead to cell apoptosis and reduced ovarian reserve.^13^ Anticancer drugs cause DNA damage, inhibition of cell division, and finally lead to cell apoptosis. In the ovary, DNA damage, such as chemotherapy‐induced DNA double‐strand breaks, activates apoptosis of somatic granulosa cells and oocytes.^14^, ^15^

Despite the enormous impact of POF on the general health and quality of life of affected women, its pathophysiology is unclear. For this reason, animal models give us the opportunity to examine the pathogenesis comprehensively and hypothetically. Therefore, several attempts have been made to introduce an optimal model for POF.

## 2 DIFFERENT METHODS OF CREATING POF ANIMAL MODELS

Currently, widely used animal models of POF can be classified as: (1) the POF model induced by chemotherapy drugs, (2) the radiotherapy‐induced POF model, (3) the genetically induced POF model, (4) the POF model induced by D‐galactose, (5) the natural ovarian aging‐induced POF model, (6) the physiological POF model with consecutive superovulation, (7) the oophorectomy POF model, (8) the POF model induced by 4‐vinylcyclohexene diepoxide, (9) the autoimmunity‐induced POF model, and (10) the psychological stress POF model, all of which are discussed in detail below.

### 2.1 POF model induced by chemotherapy drugs

#### 2.1.1 Cyclophosphamide

**FIGURE 1:**
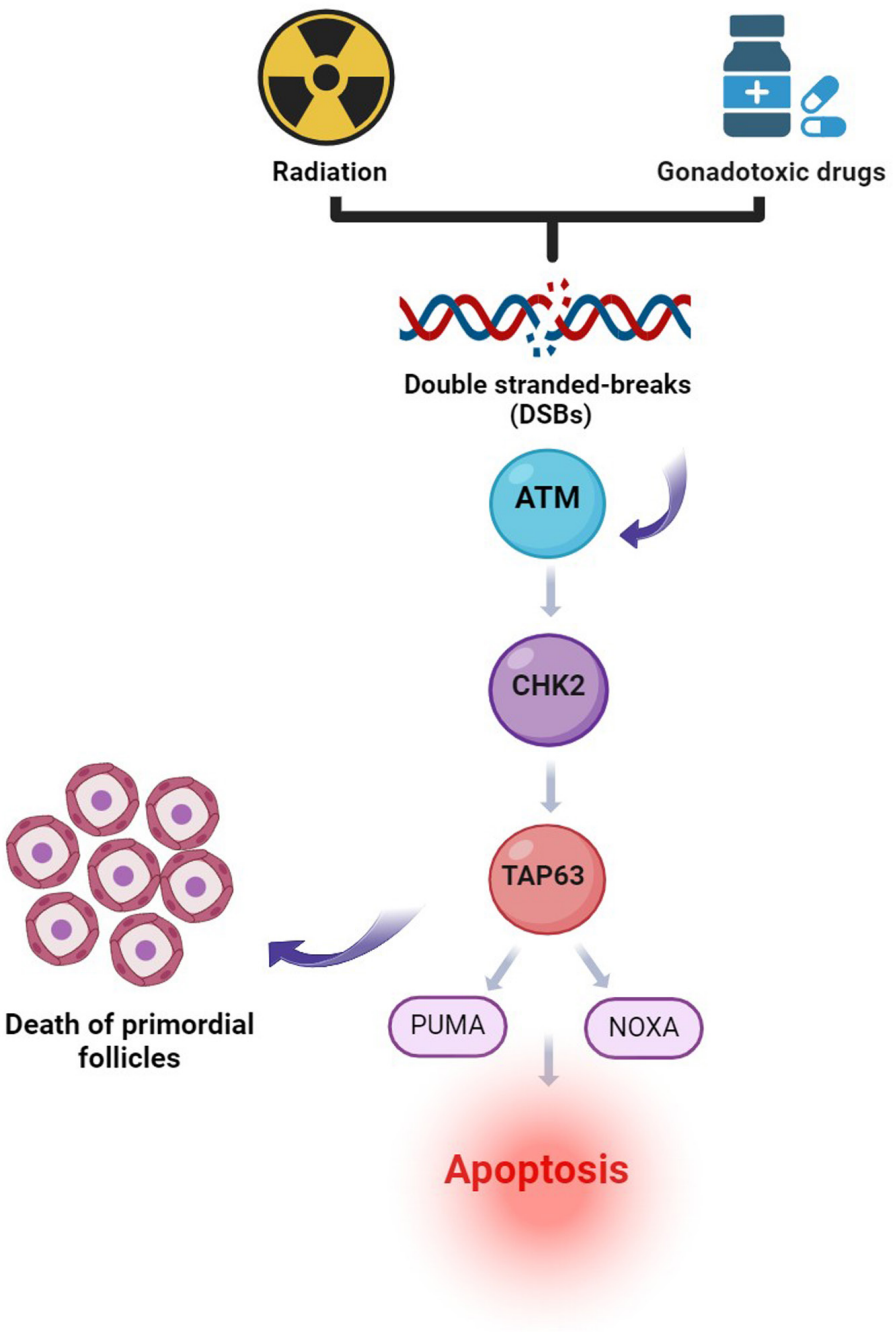
The schematic diagram for gonadotoxic drugs and radiation‐induced primordial follicle death via the TAp63 signaling pathway. Gonadotoxic drugs and radiation treatment cause DNA damage in oocytes and induce a surveillance factor, CHK2, followed by activation of NOXA, and PUMA. Therefore, DNA‐damaged oocytes undergo apoptosis. ATM, Ataxia‐telangiectasia mutated; CHK2, Checkpoint kinase 2. Created with BioRender.com.

**FIGURE 2:**
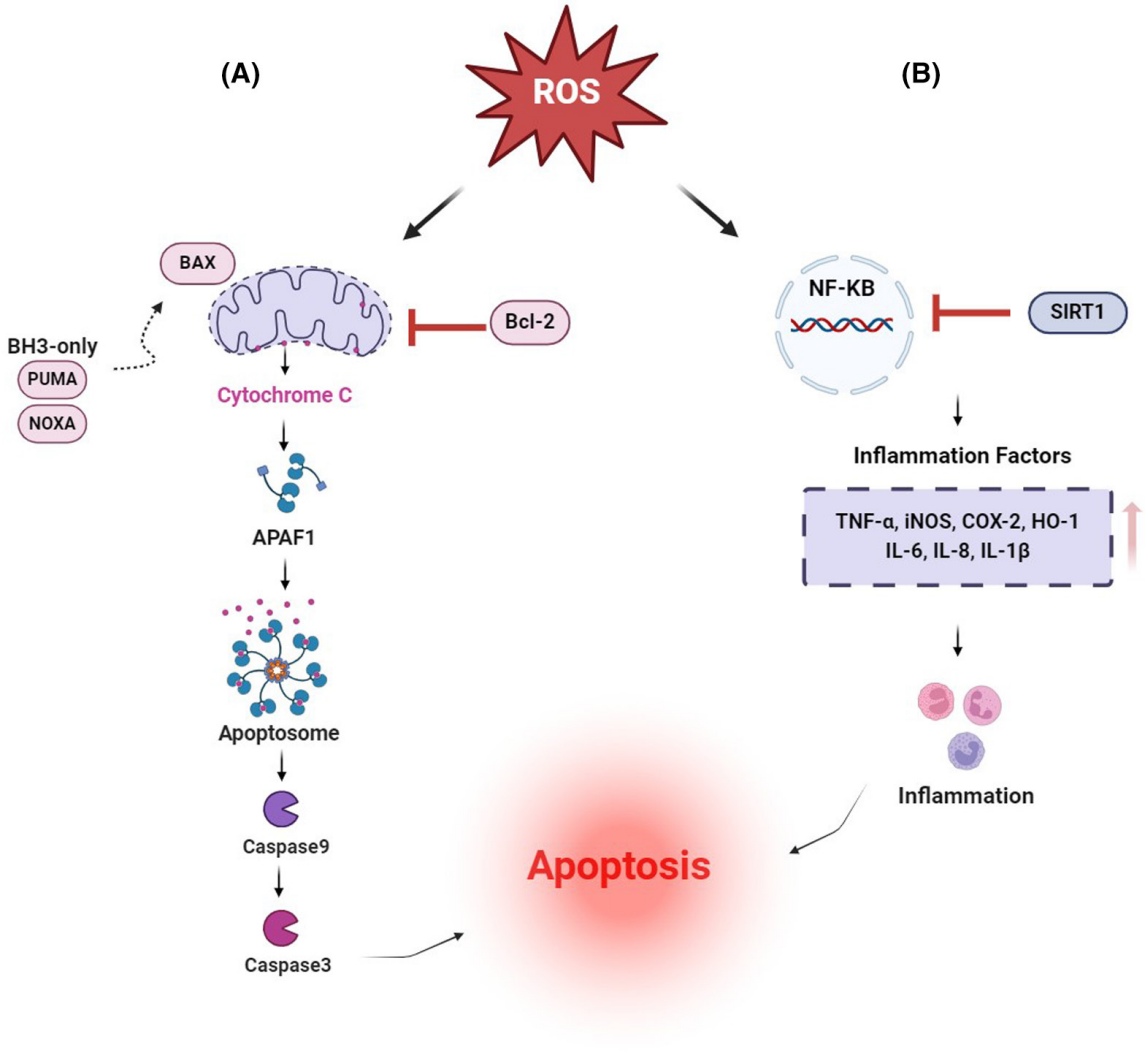
Two signaling pathways for apoptosis in premature ovarian failure via oxidative stress. (A) Activation of the mitochondrial apoptosis pathway. BAX (members of the pro‐apoptotic subgroup of the BCL‐2 family), which then assemble into large complexes that cause breaches in the mitochondrial outer membrane and the release of apoptogenic factors, such as cytochrome c. Cytochrome c, via binding to APAF1, causes the formation of the apoptosome. The initiator Caspase 9 becomes activated within this complex, which subsequently triggers the proteolytic activation of the effector Caspase 3. (B) Activation of SIRT1 pathway and inflammatory response via increased inflammatory factors. APAF1, Apoptotic peptidase activating factor 1; NF‐KB, Nuclear factor kappa B; NOXA, Phorbol‐12‐myristate‐13‐acetate; PUMA, P53 upregulated modulator of apoptosis; ROS, Reactive oxygen species; SIRT1, Silent information regulator 1. Created with BioRender.com.

**FIGURE 3:**
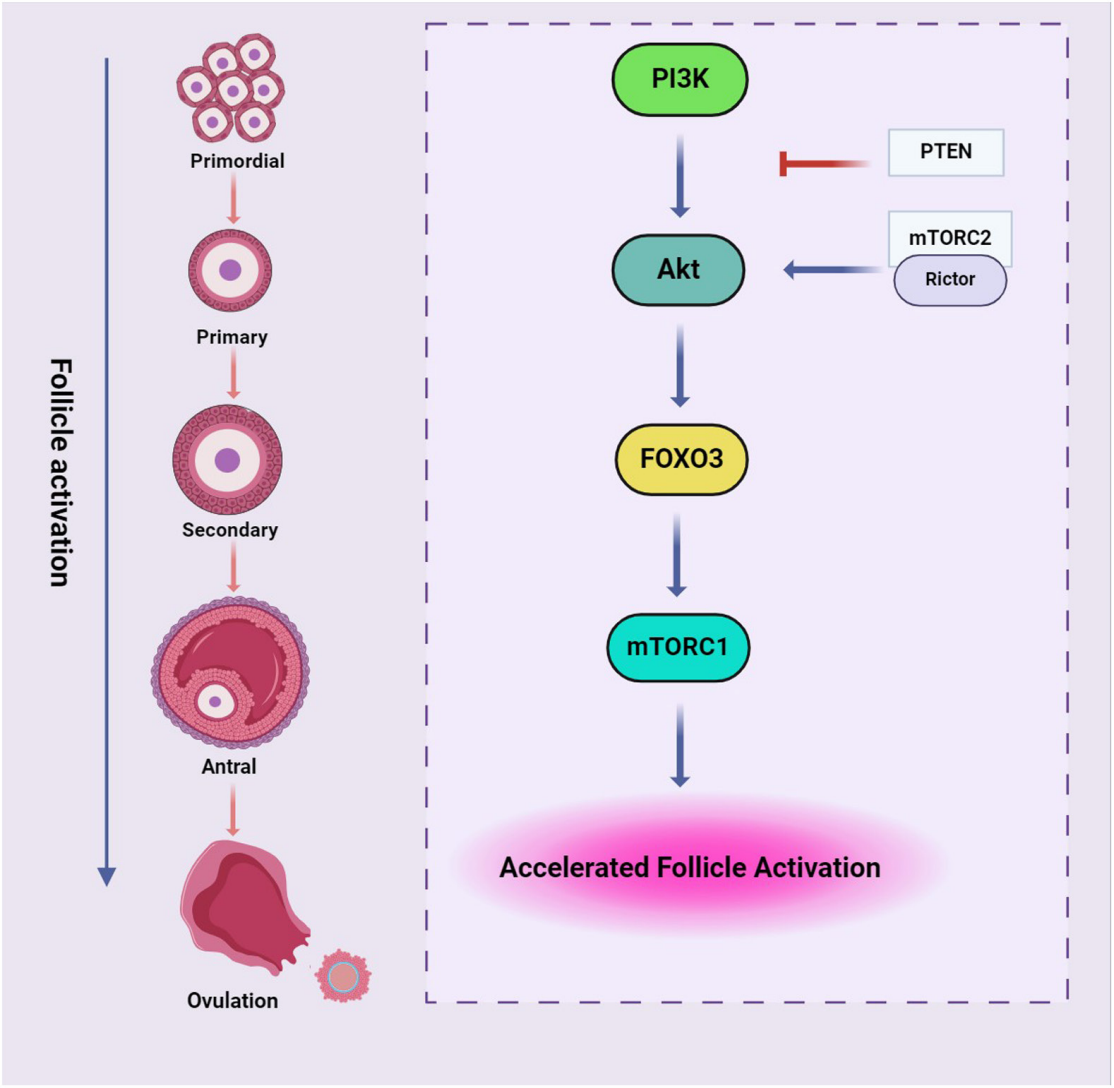
The schematic diagram for regulation of primordial follicle activation via the PI3K signaling pathway. The accelerated activation of follicles via this pathway ultimately leads to the depletion of ovarian reserve. AKT, Protein kinase B; FOXO3, Forkhead Box O3; mTOR, Mammalian target of rapamycin; PI3K, Phosphoinositide 3‐kinase; PTEN, Phosphatase and tensin homolog deleted on chromosome 10. Created with BioRender.com.

**FIGURE 4:**
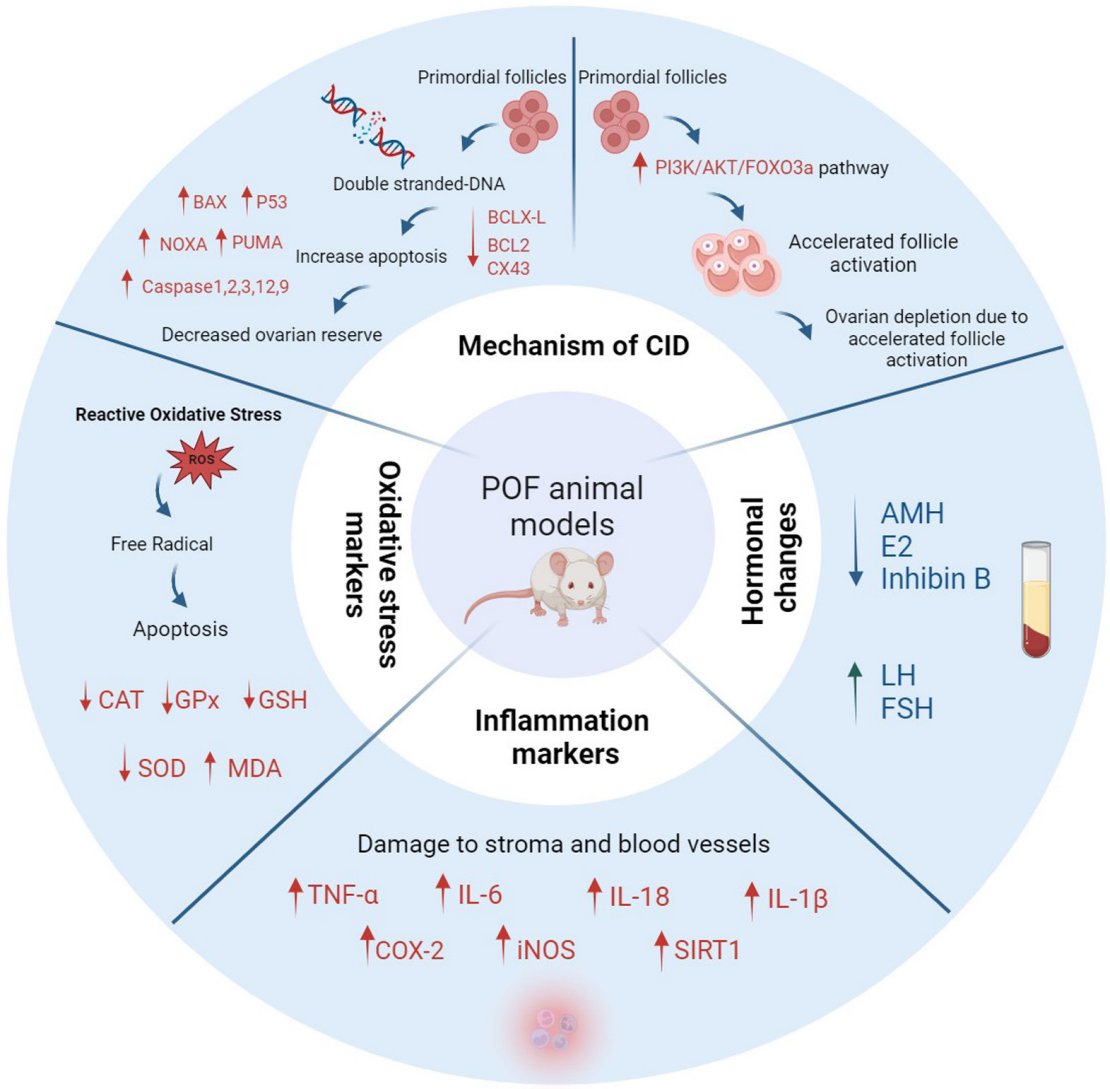
Impact of cytotoxic agents on the ovary and the molecular pathways involved in a premature ovarian failure model in murine. CAT, Catalase; CID, Chemotherapy induced damage; COX‐2, Cyclooxygenase‐2; CX43, Connexin 43 protein; GPx, Glutathione peroxidase; GSH, Glutathione; iNOS, Inducible nitric oxide synthase; MDA, Malondialdehyde; SIRT1, Silent information regulator 1; SOD, Superoxide dismutase; TNF‐α, Tumor necrosis factor α. Created with BioRender.com.

Cyclophosphamide (CTX; chemical formula C_7_H_15_Cl_2_N_2_O_2_P·H_2_·H_2_O) is an effective alkylating anticancer agent widely used in cancer patients since 1950.^16^, ^17^ CTX was the first chemotherapy drug associated with amenorrhea/POF and ovarian dysfunction (mouse and human)^16^, ^18^ and is responsible for most cases of premature ovarian failure. It is also considered one of the most important gonadotoxic agents.^19^ By accelerating the maturation process of ovarian follicles to mature follicles, CTX reduces ovarian reserves and eventually leads to premature ovarian failure in rats.^20^ Recent studies have shown that cyclophosphamide decreases AMH, E2, estrogen, and progesterone and increases FSH and LH hormones.^4^, ^21^, ^22^, ^23^, ^24^ There is also clear evidence that CTX leads to an up‐regulation of apoptosis in the ovary, as evidenced by rapid induction of DNA breaks^20^ and altered expression levels of pro‐apoptotic and anti‐apoptotic genes.^25^ Another recent study has shown that the DNA damage‐induced p53 upregulated modulator of apoptosis (PUMA) protein plays a vital role in the induction of oocyte apoptosis in rodents following cyclophosphamide treatment and that cyclophosphamide‐induced follicular loss acts through a TAp63‐independent pathway,^26^ while it has also been shown that the DNA damage and oocyte apoptosis induced by cyclophosphamide in the ovarian reserve involves the ATM‐CHK2‐TAp63 signaling pathway.^27^ Other studies using a mouse animal model have confirmed the role of this pathway in the apoptosis of primordial follicle oocytes caused by cyclophosphamide.^28^, ^29^ Recently, TAp63 (a specific isoform of P53, and a member of the P63 family) has been shown to be present in the oocyte nucleus and is a key regulator of the DNA damage or repair system in oocyte primordial follicles.^30^, ^31^ ATM kinase is activated in response to DSBs.^32^ Checkpoint kinase 2 (CHK2) is a protein component of the meiotic DNA damage checkpoint involved in DNA repair. It is a molecule downstream of ATM and subsequently transduces the signal to its downstream molecules, P53 and TAp63, and irreversibly activates the apoptotic pathway through modulator of apoptosis PUMA and phorbol‐12‐myristate‐13‐acetate (NOXA)‐induced proteins in the primordial follicles.^33^ Finally, this pathway causes the depletion of the ovarian follicle reserves and POF^34^, ^35^ (Figure 1). Previous findings have also shown that apoptosis of granulosa cells in growing follicles occurs through the activation of the mitochondrial pathway and an increase in the expression of apoptotic proteins Bax, Caspase3, Caspase1 and a decrease in the expression of the anti‐apoptotic protein Bcl‐2 and Bclx‐l^22^, ^36^, ^37^, ^38^, ^39^ (Figure 2A). CTX has also been shown to cause a decrease in the number of primordial, primary, secondary, and antral follicles, as well as an increase in atretic follicles in the ovaries of mice and rats.^21^, ^23^, ^24^, ^26^, ^36^, ^40^, ^41^ The reduction of the follicular reserve caused by cyclophosphamide is related to overstimulation and reduction of the primordial follicle (PMF) reserve through activation of the PTEN/Akt/FOXO3 signaling pathway.^23^, ^42^, ^43^ Increased phosphorylation of S473 AKT, S65 4E‐BP1 to P‐AKT, and P‐4E‐BP1 upon exposure to cyclophosphamide was shown by Goldman et al.^44^ In contrast, previous studies in a rodent model showed that inhibition of mTOR (mammalian target of rapamycin) prevented cyclophosphamide‐induced activation of PMFs through the PTEN/Akt/mTOR pathway^44^, ^45^ (Figure 3). Dysregulation of the Rictor/mTORC2/Akt/Foxo3a signaling axis can lead to cell apoptosis.^37^ Exposure to cyclophosphamide causes follicular damage and POF induced by oxidative stress (OS).^38^, ^46^ Cyclophosphamide increases the production of reactive oxygen species (ROS) and decreases enzymatic and non‐enzymatic antioxidants in mouse and rat ovarian tissue, which causes mitochondrial dysfunction, inflammation, lipid peroxidation, and apoptosis.^10^, ^23^, ^47^, ^48^, ^49^ Decreases in the levels of antioxidant enzymes such as superoxide dismutase (SOD), glutathione peroxidase (GPx) and catalase (CAT), and an increase in malondialdehyde (MDA) have been seen in premature ovarian failure caused by cyclophosphamide.^22^, ^47^, ^50^, ^51^, ^52^, ^53^ In rats treated with CTX, the drug significantly increased the expression of Nrf2 (nuclear factor erythroid‐related factor 2), a key signaling transcription factor, and decreased the expression of the ovarian HO‐1 (hemooxygenase1) gene.^10^ An essential sensor for oxidative stress is silent information regulator 1 (SIRT1), which coordinates cellular defense and repair mechanisms, controls the cell fate, and prevents the survival of damaged cells.^54^ SIRT1 acts in both inflammation and oxidative stress by inhibiting nuclear factor kappa B (NF‐kB) signaling and stimulating the antioxidant response through the expression of the FOXO transcription factor. Cyclophosphamide increases SIRT1 in mouse oocytes as an adaptive response to oxidative stress.^53^, ^55^, ^56^, ^57^ In rats treated with CTX, increased levels of NF‐kB, tumor necrosis factor α (TNF‐α), and cyclooxygenase 2 (COX‐2), and gene expression of inducible nitric oxide synthase (iNOS) and caspase 3 were observed^22^ (Figures 2B and 4).

#### 2.1.2 Cisplatin

Platinum‐based chemotherapy agents, including carboplatin, cisplatin, lobaplatin, nedaplatin, and oxaliplatin, account for approximately 50% of anticancer drugs used in the clinical setting.^58^ The most common platinum‐based drug is cisplatin (CIS; chemical formula Cl_2_H_6_N_2_Pt).^59^ The ability of CIS to inhibit cell division was first recognized in the 1960s^60^ and it became the first platinum compound approved by the FDA for cancer treatment in 1987. Cisplatin is a heavy metal compound and acts as a DNA cross‐linking agent that interferes with DNA repair mechanisms, blocks cell division, causes DNA damage, and induces apoptotic cell death. CIS interacts with several different cellular components, but its primary biological target is DNA.^16^ Exposure of mouse and rat ovaries to CIS is associated with loss of ovarian reserve and increased follicular atresia.^61^, ^62^, ^63^ Cisplatin‐induced DNA damage is proposed to lead to the activation of the apoptotic pathway through the activation of the apoptotic gene Bax and Caspase3 and inflammatory response^64^ (Figure 2A,B). The tumor suppressor protein p53 and its family members, which play an important role in the extent of CIS‐induced DNA damage, are repaired and ultimately determine the cell's fate. ATR, a kinase involved in checkpoint activation, is activated by CIS and can, in turn, activate p53 as well as specific pathways of the MAPK cascade.^65^ Both mouse and human primordial oocytes contain high concentrations of TAp63. Cisplatin activates TAp63α through the ATR/CHK1/CK1 pathway, and TAp63α, in turn, increases the expression of BH3‐only proapoptotic proteins PUMA and NOXA, which ultimately leads to oocyte apoptosis^66^ (Figure 2). Studies show that the exposure of primordial follicles to CIS causes over‐activation of PMFs and reduced ovarian reserve and ultimately causes POF by activating the PTEN/Akt/FOXO3 pathway^67^, ^68^, ^69^, ^70^ (Figure 3). Cisplatin significantly increases oxidative stress and decreases the level of SOD, GSH, and GPx antioxidants in rat ovarian tissue^46^, ^71^

#### 2.1.3 Doxorubicin

Doxorubicin (DOX), with the chemical formula C_27_H_29_NO_11_, is an anthracycline antibiotic (14‐hydroxydaunomicyn), also known by the brand names Adriamycin or Rubex, which interferes with DNA and prevents its replication and transcription and is used to treat a wide range of cancers including breast, lung, lymphoma, gastric, ovarian, and leukemia. Recent evidence suggests that it is moderately gonadotoxic. The primary and best‐known mechanism of DOX is inhibition of the nuclear enzyme topoisomerase II.^72^, ^73^, ^74^ DOX prevents the formation of the topoisomerase II‐DNA complex after the cleavage step by accumulating DNA fragments, which ultimately causes cell death. In addition, DOX stimulates the production of oxygen free radicals and other reactive oxygen species and also causes mitochondrial dysfunction.^75^, ^76^ Both free radical production and mitochondrial damage negatively affect cellular metabolism and membrane integrity, leading to severe side effects. DOX also enters DNA and disrupts DNA and RNA replication and protein synthesis.^77^ Granulosa cells, stromal/theca cells isolated from developing mouse follicles, and oocytes undergo double‐stranded DNA breaks after DOX injection.^78^, ^79^ Apoptosis is the main and certainly the most studied process of DOX‐induced cell death in the ovary. Previous studies have shown that apoptosis and follicular deterioration occur 12 h after DOX injection in mice.^80^, ^81^ Doxorubicin increases the induction of apoptosis of granulosa cells, increases ROS, and decreases the potential of the mitochondrial membrane. The downstream effectors in the apoptosis pathway, the death regulatory genes BAX, BCL‐2, and p53 are associated. When granulosa cells are exposed to DOX in vitro, they show increased p53 mRNA expression^82^ (Figure 2A). According to the results of these studies, doxorubicin activates mitochondrial apoptosis through Caspase12.^83^ DOX can also cause DNA damage in relatively larger follicles, such as primary, secondary, and antral follicles, with many granulosa cells.^79^ As mentioned above, Tap63 is activated after oocyte DNA damage, and DOX affects Tap63, providing a pathway for the loss of PMFs^66^ (Figures 1 and 4).

#### 2.1.4 Busulfan

Busulfan (BU) is a drug combined with cyclophosphamide for managing and treating allogeneic hematopoietic progenitor cell transplantation, especially for patients with chronic myelogenous leukemia. This drug, which belongs to the group of anticancer drugs and is defined as an alkylating agent, has been used since the 1950s. By binding to cysteine molecules of histone proteins, busulfan leads to DNA binding to proteins. Busulfan also disrupts the cellular redox balance by interacting with the sulfhydryl groups of glutathione, thereby increasing oxidative stress in cancer cells.^84^ The classical chemotherapy drugs cyclophosphamide and busulfan (CTX/BU), often used to treat cancer in the clinic, have severe reproductive toxicity and have been used to induce POF in an animal model.^85^

The combination of busulfan and cyclophosphamide decreases the size of ovaries, decreases the number of follicles,^86^ and increases the number of atretic follicles in mice. On the other hand, busulfan and cyclophosphamide cause apoptosis by increasing the pro‐inflammatory cytokines IL‐6 and IL‐8, reducing the antioxidant factors superoxide dismutase and Nrf2, as well as reducing the anti‐apoptotic protein Bcl‐2 and increasing the apoptotic protein Bax^85^, ^87^, ^88^ (Figure 1A,B). In this study, it has been shown that the weights of the ovaries and the uterus, as well as the body weight, were significantly reduced under treatment with cyclophosphamide and busulfan.^89^, ^90^ Liu et al. showed that in mice treated with CTX and BU, increased expression of AKT and PI3K phosphorylation occurs^87^ (Figures 3 and 4).

### 2.2 Radiotherapy‐induced POF model

Radiotherapy is one of the standard cytotoxic therapies for cancer. However, it has a profound effect on ovarian function, leading to premature ovarian failure and infertility.^91^ Radiation to the brain can damage the pituitary gland and lead to the deregulation of gonadotropin hormones, which are needed to maintain normal ovarian function.^92^ In addition, radiation directly destroys ovarian follicles, leading to depletion of the primordial pool,^93^ thereby predisposing women to premature menopause and infertility,^94^ during or shortly after completion of irradiation.^91^ Many molecular mechanisms are involved in the pathogenesis of radiation‐induced toxicity, and one of these critical mechanisms is the inflammatory cascade.^95^ Ionizing radiation reaching cancerous tissue produces toxic free radicals and reactive oxygen species that attack critical macromolecules such as DNA, disrupt mitochondrial membrane integrity, and activate the inflammatory and apoptotic pathways, leading to follicular death^96^, ^97^ (Figure 2A,B). Several studies have shown that DSBs induced by radiation induce apoptosis and follicle pool depletion through activation of the ATM/CHK2/Tap63 pathway^34^, ^98^, ^99^, ^100^ (Figure 2).

The estimated dose at which half of the follicles are destroyed in humans (LD_50_) is 4 Gy.^101^ In another study, a significant decrease in antral follicle count (AFC) and the weight of the ovaries of rats and a decrease in the serum levels of AMH and E2 were observed 2 days after irradiation. On the other hand, oxidative stress caused by gamma radiation increased in rat ovaries and caused a significant decrease in antioxidant activities.^102^ Ovarian damage caused by irradiation causes an increase in inflammation by increasing the inflammatory factors NF‐kB, TNF α, iNOS, and COX‐2 and increasing apoptosis by increasing the expression of cytochrome c and caspase 3.^103^ Exposure to ϒ radiation decreases FOXO1 and FOXO3 expression, which could reflect early activation of primordial follicles followed by secondary follicular atresia. In addition, a significant increase in apoptotic factors such as Bax and Bax/Bcl2 ratio and caspase‐3 expression was observed in rats exposed to radiation (Figures 1 and 4).

### 2.3 Genetically induced POF model

Genetic analyses of POF patients have identified several chromosomal abnormalities, single gene mutations, and genetic polymorphisms from different biological pathways associated with the development of POF. However, the genetic defects investigated so far have been shown to contribute only a small percentage to the development of POF. The diverse etiology of POF is consistent with these findings and suggests that the pathogenesis of non‐syndromic POF is unlikely to be caused by a single gene or genetic defect, but rather that POF is a heterogeneous genetic disease involving the interaction of multiple genetic defects and environmental factors.^104^ Abnormalities of the X chromosome have long been recognized as the most common genetic cause of POF, accounting for about 12% of cases. These abnormalities usually include complete or partial X chromosome deletions, duplications, or translocations.^105^, ^106^ Important genes associated with POF and identified on the long arm of the X chromosome include the fragile x mental retardation 1 (FMR1) gene, which is associated with fragile X syndrome^107^ Apart from chromosomal abnormalities, single gene changes can also cause POI.^108^

#### 2.3.1 Genes related to meiosis and DNA replication and repair

If an error occurs during meiosis, DNA replication, or DNA repair, the genetic information is negatively affected and leads to apoptosis of germ cells and infertility. Therefore, investigating the genes involved in these processes is useful for a better understanding of POF. Recent studies have shown that SPO11 (sporulation protein), DMC1 (disrupted meiotic cDNA 1 homolog), MSH4 (mutS homolog 4), and MSH5 (mutS homolog 5) are important for the formation of this disease.^108^, ^109^, ^110^ Meiotic events play an essential role in the formation of follicles, and mouse models caused by mutations in meiosis‐related genes show defects in oocyte development. SPO11 is an essential enzyme for creating DSBs during meiosis.^111^ Spo11^−/−^ mice have a decreased number of primordial follicles at the time of follicle formation, leading to complete infertility in adults due to the lack of mature follicles.^112^ Msh4^−/−^ and Msh5^−/−^ mice are sterile and lack oocytes due to defects in meiosis and severe gametogenic failure.^113^

#### 2.3.2 Genes related to the survival, growth, and activation of primordial follicles

FIGLA (Factor in the germline, alpha), NOBOX (Newborn ovary homeobox‐encoding gene) and FOXL2 (Forkhead box L2) genes are important for the transition of primordial follicles to primary follicles. FOXL2 is mainly expressed in granulosa cells in ovaries from embryogenesis to adult life.^114^ In Foxl2^−/−^ ovaries, granulosa cell differentiation is blocked and thus the transition from primordial follicles to primary follicles is blocked. Mice are infertile due to impaired follicular development.^115^ Mutations in the FOXL2 gene cause blepharophimosis/ptosis/epicanthus inversus syndrome (BPES) type I associated with POF.^114^, ^115^ The NOBOX gene, an oocyte‐specific homeobox gene, is essential for follicle development after birth. In mice lacking the NOBOX gene, accelerated oocyte loss occurs after birth and impaired transition of primordial follicles to developing follicles.^116^ FIGLA is expressed in female germ cells and plays an important role in oogenesis. In FIGLA‐deficient mice, although embryonic gonadogenesis appears normal, primordial follicles fail to form at birth, and a severe reduction in oocytes results in shrunken ovaries and sterility in female mice.^117^ Estrogen deficiency causes significant metabolic changes in mice due to loss of FSH‐R signaling and subsequent cessation of reproductive function. The FSHR^−/−^ mouse phenotype is a suitable model for studying ovarian failure in humans [13]. In one study, FSH‐R knockout mutations (FORKO) in mice caused disturbances in the estrous cycle, ovulation, and uterine atrophy, as well as ovarian dysfunction and decreased estrogen.^118^, ^119^ In another study of adult female FORKO mice, ovaries were 45% smaller and increased levels of testosterone and LH hormones were observed.^118^

#### 2.3.3 Genes related to the development of primordial germ cells (PGCs)

Defects in the development of PGCs lead to the lack of functional eggs and follicles and as a result POF.^109^ BMPs, members of the TGF family of growth factors, are important for the migration and development of PGCs. BMP4^−/−^ embryos lack PGCs.^120^ Also, the number of PGCs is significantly reduced in BMP8b^−/−^ and BMP2^−/−^ mice, which leads to the reduction or loss of primordial follicles.^121^ DAZL (Deleted in azoospermia‐like) gene is necessary for the growth of germ cells.^122^ Disruption of the DAZL gene leads to a decrease in the number of germ cells in mouse embryos and the complete absence of ovarian follicles in mature mouse ovaries.^123^ Nanos C2HC‐Type Zinc Finger 3 (NANOS3) gene is known to be required for PGC development, migration, and maintenance. Female mice lacking the NANOS3 gene are infertile because they don't have any PGCs in the gonad by embryonic day 12.5 (E12.5).^124^

#### 2.3.4 Genes related to RNA metabolism and translation

Defects in mitochondrial function and non‐coding (small) RNAs, ncRNAs, and long ncRNAs are useful for doctors to diagnose idiopathic POI cases and predict the risk of POI in women.^108^ Eukaryotic translation initiation factor 4E nuclear import factor 1 (EIF4ENIF1) is an RNA‐binding protein and is a component of the cytoplasmic ribonucleoprotein complex such as the mitochondrial‐associated ribonucleoprotein domain (MARDO).^125^ EIF4ENIF1 is one of the genes known to cause POF and plays an essential role in inhibiting mRNA translation and regulating mRNA instability in ovarian cells.^126^ In one study, it was shown that haploinsufficiency of EIF4ENIF1 protein causes reproductive damage in mice and impairs oocyte maturation.^127^

POF is a complex disorder that may have several different genetic causes.^109^ The genetic model is a complex model in which the phenotype of complete gene deletion in knockout models may not be mimicked by single gene mutations.^128^ The disadvantage of this POF animal model is the high cost.^129^ The genes associated with POF are classified according to the biological processes they participate in (Table 1).

**TABLE 1:** List of candidate genes associated with non‐syndromic premature ovarian failure.

Classification	Gene	Mechanism of action	Reference
Meiosis and DNA replication and repair	SPO11	Creating DSBs during meiosis	[111]
	MSH4	Synapsis and DNA repair	[130]
	MSH5	Synapsis and DNA repair	[130]
Survival, growth and activation of primordial follicles	FIGLA	Oogenesis and development of secondary follicles	[131]
	NOBOX	Folliculogenesis and steroidogenesis	[116]
	FOXL2	Growth and development of follicles	[115]
	FSHR	Follicular development and ovarian hormone regulation	[132]
Development of PGCs	BMPs	Generation of primordial germ cells	[120]
	DAZL	Growth of germ cells	[122]
	NANOS3	Migration, development, and maintenance of PGCs	[124]
RNA metabolism and translation	EIF4ENIF1	Translation repression	[127]

### 2.4 POF model induced by D‐galactose (D‐gal)

Galactosemia – a rare genetic metabolic disorder that affects a person's ability to metabolize the sugar galactose – is associated with POF. Accumulated galactose (GAL) in the absence of galactose‐1‐phosphate uridyltransferase (GALT) has an ototoxic effect in galactosemic women, ultimately leading to accelerated decline in the ovarian follicle reserve and occurrence of POF.^133^, ^134^ The ovotoxicity characteristic of galactose has led to using this monosaccharide to induce POF modeling. According to the results of these studies, D‐galactose significantly increases the expression of γH2AX and BP153, two markers of DNA damage.^135^ Also, an increase in the level of FSH and LH hormones and a decrease in the level of E2, AMH, and progesterone hormones have been observed in rats treated with D‐galactose.^135^, ^136^, ^137^, ^138^ The results of a study in rats show that the ovarian dysfunction in galactosemia may be due to the higher level of expression of cell death inducers (Fas/FasL) and decreased expression of survival signals (Riap and Xiap) in developing follicles, resulting in apoptosis.^139^ Treatment with D‐gal reduced the ratio of primordial follicles, primary follicles, and antral follicles and increased the ratio of atretic follicles,^56^, ^135^, ^136^ while, on the other hand, it decreased the activity of antioxidant enzymes SOD, GSH, and decreased Nrf2 expression. The downstream antioxidant enzymes in treated mice are GCLC, HO‐1, and NQO1.^56^, ^136^, ^137^ Decreased levels of SOD, GSH‐px, and increased levels of MDA,^137^, ^138^ mRNA Sod2, and mRNA CAT, as well as increased levels of Bax, Caspase 3, Caspase 9, and decreased levels of Bcl‐2, p‐AKt, p‐PI3K in mice treated with D‐gal have been observed^136^, ^137^, ^138^ (Figures 1A and 3). In a study by Lee et al., an increase in the level of inflammatory cytokines such as IL‐6, TNF‐α, and IL‐1β was observed in the ovaries of rats treated with D‐gal^138^ (Figure 2B). Although the GAL animal model can simulate the physiological aging characteristics of clinical POF patients, this model has a lower success rate and a longer cycle^140^ (Figure 4).

### 2.5 Natural ovarian aging‐induced POF model

For humans, especially those who have passed their 40s, the process of ovarian follicle decline can be accelerated by many factors such as aging.^141^ After birth, the quality and quantity of oocytes in the ovarian cortex follicles gradually decrease during normal ovarian aging (NOA).^142^ In addition, with the aging of ovaries, inflammatory cytokines (TNF‐α, IL‐6) and oxidant factors increase, so oxidative stress and inflammation play a role in the pathogenesis of POF^143^ (Figure 2B). In several studies, female rats (12–15 months old) were used for the natural ovarian aging‐induced POF model (NOA‐POF). A decrease in the level of estradiol hormone, AMH, an increase in the level of FSH hormone, and an increase in oxidative stress and apoptosis were observed in NOA mice.^144^, ^145^, ^146^ One of the natural ovarian aging‐induced POF model's disadvantages is that it requires a considerable amount of time. On the other hand, there are also some advantages including low cost, and not having any side effects since no drugs are injected (Figure 4).

### 2.6 Physiological POF model with consecutive superovulation

Induction of POF in mice using 5 to 15 consecutive superovulation treatments with a continuous intraperitoneal injection of pregnant mare serum (PMSG), human chorionic gonadotropin (HCG), and prostaglandin F2α (PGF2α) resulted in a stable physiological model. According to studies by Xiaowei Nie et al., the sequential treatment of superovulation causes a significant increase in the level of FSH and LH hormones, the oxidative stress indicator MDA, and the number of antral and atretic follicles, as well as a significant decrease in the level of E2, P and Inhibin B hormones. SOD, GSH‐Px enzymes, and the number of primordial, primary and secondary follicles. Increased apoptosis of mouse ovarian granulosa cells through the SIRT1/FOXO1 signaling pathway was observed in the five consecutive superovulation treatment groups^147^ (Figures 2B and 4).

### 2.7 Oophorectomy POF model

Oophorectomy is the surgical removal of one or both ovaries. It may be done for ovarian pathology (such as neoplasm), risk reduction purposes (reducing the risk of reoperation or cancer), or fertility preservation.^148^, ^149^, ^150^ Bilateral oophorectomy causes infertility and menopause in patients with physiological changes.^151^ In one study, an oophorectomy was created in a rat by removing one of the ovaries (ovariectomized). As a result of this study, the serum levels of AMH and E2 hormones decreased significantly in the oophorectomy rat model. Also, this model's total number of primordial follicles decreased, and ROS levels increased compared to the control group^152^ (Figure 4). Ovariectomy has been a valuable tool for understanding estrogen deficiency through animal experiments. The disadvantage of oophorectomy model is that it cannot mimic spontaneous POF because the transitional period of ovarian emptying is neglected.^133^

### 2.8 POF model induced by 4‐vinylcyclohexene diepoxide

4‐Vinylcyclohexene diepoxide (VCD) is a metabolite of 4‐vinylcyclohexene (VCH), which is often used in the industrial context and is classified as a carcinogen according to the classification of the International Agency for Research on Cancer (IARC). VCD, a chemical intermediate, is a reactive diluent for epoxy resins and diepoxides. In the studies conducted by the National Toxicology Program (NTP) on the carcinogenic potential of VCD, it was reported that in addition to carcinogenic effects, it also has ovotoxic effects.^153^ Exposure to commercial or environmental toxins can cause serious fertility problems in women. Today, one of the biggest causes of infertility is the increasing exposure to these toxic agents. The toxic effect of VCD can induce premature ovarian failure by reducing the primordial follicle reserve in the ovaries.^154^ In vivo and in vitro experiments have shown that VCD can cause oocyte dysfunction and toxic effects in the ovary.^155^, ^156^ According to the results of previous studies, Bax, Caspase2, and Caspase3, but not acid sphingomyelinase (ASMase) or aromatic hydrocarbon receptor (Ahr), are functionally important in VCD‐induced follicle loss.^156^ Also, in this study, VCD caused a decrease in all types of follicles, especially primordial and primary follicles, as well as a decrease in AMH hormone expression in rat ovaries.^153^ Gap junction connexin 43 protein (Cx43) is an inversely related protein in apoptosis and plays a vital role in the survival of granulosa cells.^157^ In this study,^153^ by increasing the expression of Caspase 3 and iNOS, the increase in oxidative stress caused by exposure to VCD caused a decrease in the expression of Cx43 protein (which plays an important role in the survival of granulosa cells and has an inverse relationship with the apoptosis index) and an increase in apoptosis (Figures 2A,B and 4).

### 2.9 Autoimmune‐induced premature ovarian failure

Although the exact causes of POF remain unknown, autoimmune mechanisms may be involved in approximately 10–30% of women with POF,^158^, ^159^ which may be a simple autoimmune disease of the ovary or associated with other immune disorders. The immune pathology of autoimmune ovarian disease (AOD) is mainly associated with non‐infectious ovarian inflammation (oophoritis), ovarian atrophy, and serum autoantibodies to ovarian antigens.^160^ Ovarian dysfunction caused by an autoimmune mechanism may be related to zona pellucida (ZP) antigens such as ZP3 glycoprotein.^161^ ZP3 is the main receptor in the zona pellucida that binds to sperm during fertilization.^162^ In one study, an animal model of POF in mice was created by injecting glycoprotein ZP3 to cause autoimmune damage to the ovary.^158^ Immunization of animals with ZP3 glycoprotein by activating T cells and anti‐ZP3 antibodies (AZPAbs) interferes with normal follicular development and leads to follicular emptying and amenorrhea.^158^, ^163^ Evaluation of AZPAb expression, examination of ovarian histopathology, and a significant increase of FSH hormone and a decrease of E2 hormone show that the POF model was successfully established through this approach^158^ (Figure 4). In another study, POF was induced in rats by causing autoimmune ovarian inflammation by subcutaneous injection of 0.35 mL of ovarian antigen 3 times, once every 10 days. Rat ovarian tissue supernatant protein with Freund's adjuvant acts as ovarian antigen. Decreased levels of E2, P4, and AMH hormones and irregular estrous cycles were shown 2 weeks after ovarian antigen injection in rats.^164^ Because this method is rarely used, it is unclear what concentration of ovarian antigen can be used to successfully construct an autoimmune POF model. On the other hand, although autoantibodies are generated in most autoimmune diseases, the specific ovarian antigens involved in human autoimmune premature ovarian failure have not yet been identified.^140^, ^165^ Neonatal thymectomy in mice can create a disease with symptoms similar to human POF [10, 11]. Data from Tang et al.'s study show that removal of thymus from 2‐ to 4‐day‐old mice with autoimmune oophoritis results in complete loss of oocytes and follicles in adult mice [9]. When B6A mice are thymectomized 3 days after birth, 90% develop autoimmune euphoritis and premature ovarian failure.^166^, ^167^ However, removing the thymus from newborn mice is difficult and has a high mortality rate.^140^

### 2.10 Psychological stress POF model

**FIGURE 5:**
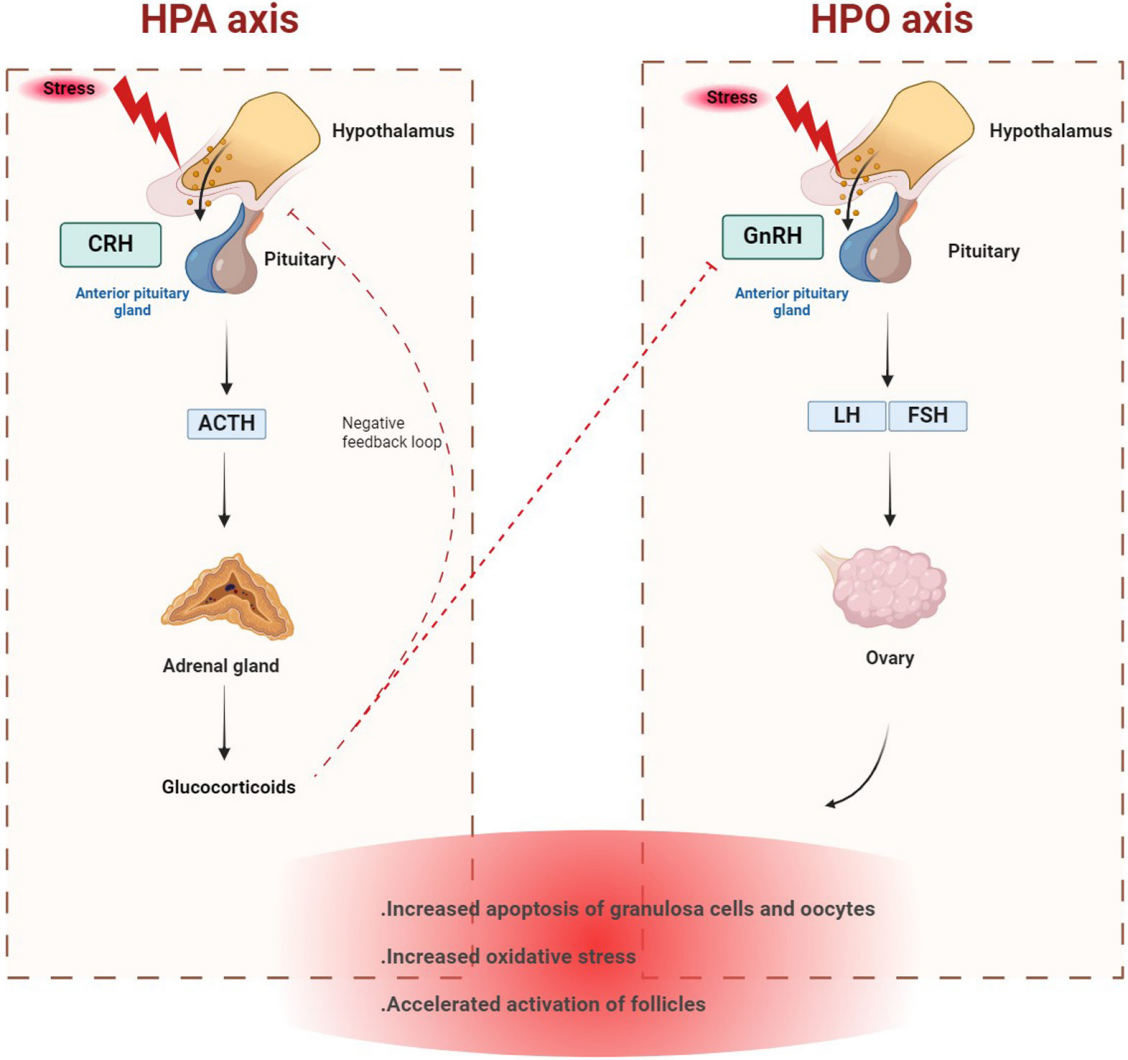
Psychological stress regulates ovarian function through the HPA and HPO axes. HPA axis: Stress exposure activates CRH and ACTH in the pituitary, which subsequently stimulates glucocorticoid secretion from the adrenal cortex. Glucocorticoids, acting via a negative feedback loop, can disrupt ovarian function by suppressing the synthesis and release of GnRH in the hypothalamus. HPO axis: GnRH released by the hypothalamus enters through the pituitary portal venous system, triggering the release of FSH and LH. Psychological stressors and disturbances in the path of these axes through oxidative stress, apoptosis of granulosa cells and oocytes, and excessive activation of primary follicles cause a decrease in ovarian function. ACTH, Adrenocorticotropic hormone; CRH, Corticotropin‐releasing hormone; GnRH, Gonadotropin‐releasing hormone; HPA, Hypothalamic–pituitary–adrenal axis; HPO, Hypothalamic–pituitary‐ovarian axis. Created with BioRender.com.

Psychological stress, for example, chronic anxiety, sadness, fear, and other negative emotions, can lead to POF and hypothalamic–pituitary‐ovarian (HPO) axis dysfunction by changing the function of the hypothalamus‐pituitary target gland axis. Failure to regulate the hypothalamic–pituitary‐ovarian axis feedback disrupts the balance of the neuroendocrine‐immune biomolecular network and ultimately leads to POF. An animal model of POF stress can be induced by alternately applying different frequencies of electro‐acoustic stimulation.^7^ Mechanistically, when people experience psychological stress, the hypothalamic–pituitary–adrenal (HPA) axis is activated, leading to the release of high levels of corticotropin‐releasing hormone (CRH) in the hypothalamus and synthesizing and secreting adrenocorticotropic hormone (ACTH) in the pituitary, which subsequently leads to glucocorticoid release into the bloodstream. The gonadotropin‐releasing hormone (GnRH) released by the hypothalamus enters through the pituitary portal venous system, triggering the release of FSH and LH.^168^ Glucocorticoids, responsible for regulating the HPA axis via a negative feedback loop, can disrupt ovarian function by suppressing the synthesis and release of GnRH in the hypothalamus, which leads to a decrease in the production of LH and FSH in the pituitary gland and terminates the stress response.^169^, ^170^ The HPA axis activation inhibits the normal functioning of the HPO axis through endocrine, paracrine, and neurological mechanisms.^171^ As a result, psychological stress can cause changes in reproductive endocrine glands. As well as being involved in the stress response, the reproductive endocrine system is vulnerable to damage caused by psychological stress and can disrupt the neuroendocrine‐immune network.^7^ Chronic unpredictable mild stress (CUMS) induces psychological stress and decreases ovarian reserve in female rats. In addition, granulosa cells underwent more apoptosis in response to psychological stress.^165^ The decrease in ovarian function is commonly attributed to oxidative stress, follicular atresia, and excessive activation of primordial follicles. The apoptosis of ovarian granulosa cells and oocytes is the primary reason for follicular atresia. Psychological stress‐induced POF is closely tied to these mechanisms.^171^ Previous studies have established that, in the ovaries of depressed‐looking mice, more granulosa cell apoptosis occurs and Bcl2 expression in granulosa cells mitochondria is significantly reduced.^172^ Oxidative stress induced by psychological stressors can lead to damage to the ovaries by raising levels of ROS. Stress can decrease the levels of antioxidant enzymes, resulting in a notable reduction in the activities of SOD, GPX, and CAT in the preantral follicles^173^ (Figure 5). The advantages of this modeling approach are that it is consistent with the known main causes of human POF and that the pathogenic pathways and pathological changes are similar to clinical observations.^140^

## 3 DISCUSSION

Many studies have used animal models of POF to investigate and identify the mechanisms of POF and develop therapeutic agents. Inducing an animal model of premature ovarian failure using a gonadotoxic agent without serious damage to other organs is challenging. However, understanding the mechanism of POF development is vital for the clinical treatment of this disease, and considering the limitations to conducting comprehensive studies in humans, animal models such as mice and rats are a powerful tool for finding the pathogenesis of this disease. In this article, we have reviewed the studies that used murine POF models. Mice and rats are the most common animal models used in laboratories to study POF^174^ because their estrous cycle is similar to that of humans, though considerably shorter. Ninety‐nine per cent of mouse genes are homologous to human genes, and there is a high degree of similarity in ovarian development processes and functions, as well as in regulation of the genetic pathway responsible for POF, between mice and rats and humans.^174^, ^175^

To ensure the establishment of an animal model of POF, the evaluation indicators mainly include hormonal analysis, histological assessment (ovarian weight, follicle count, ovarian reserve, estrous cycle, histopathology), and fertility index.

Measurement of hormonal levels, such as FSH, LH, AMH, and estradiol, is an important evaluation index for animal models of POF. FSH and LH levels typically increase in POF, while estradiol and AMH levels decrease. Low AMH indicates a decrease in ovarian reserve function.^148^ These hormonal changes can be measured in the serum or plasma of animals and can provide important insight into the pathogenesis of POF.^84^, ^176^, ^177^

Ovarian histology is an essential evaluation index of POF in animal model. Ovarian reserve refers to the primordial follicles in the ovarian cortex.^148^, ^177^ Ovarian reserve tests are performed by direct or indirect evaluation of the reduction in the number of primordial follicles, AFC and AMH.^40^, ^178^ Histological evaluation includes ovarian volume and weight, corpus luteum number, length of the estrous cycle, number of follicles, ratio of ovulation, and abnormal ovulation.^146^ In the ovarian tissues of the POF animal model, the volume and weight of the ovary are reduced, the corpus luteum, the ratio of the number of ovulation and normal ovulation is lower, and the estrous cycle is prolonged. In addition, primordial follicles, secondary follicles, and antral follicles are reduced, but atresia and apoptotic follicles are increased.^179^

Infertility in POF is caused by decreased ovarian reserve function. Fertility is, therefore, a critical evaluation index of POF animal models. Animal models of POF are often evaluated for their ability to conceive and produce offspring. The fertility of POF animals can be assessed by mating them with fertile males and monitoring their pregnancy outcomes (number of pups, pregnancy rate, litter size, abortion, stillbirth).^146^, ^180^, ^181^ Fertility assessment is an essential evaluation index of the POF animal model, as it provides information on the reproductive performance of the animals.

Using these evaluation indices, researchers can better understand the pathogenesis of POF and develop more effective treatments for this complex condition. Consequently, it is critical to evaluate animal models of POF to ensure that they accurately represent the human condition and can be used to develop effective treatments. On the other hand, the main issue in the POF condition is whether the condition will be reversible after the appearance of the POF. Spontaneous pregnancy is extremely rare in patients with POF. Women who experience POF have menstrual irregularities that interfere with their fertility. Some patients with idiopathic POF show intermittent ovarian function and hence, their chance of spontaneous pregnancy and having an uneventful pregnancy is approximately 5% [1]. Depending on whether the amenorrhea is primary or secondary, POI is reversible. Primary amenorrhea is more serious than secondary amenorrhea, and the latter is easier to reverse. The results of laboratory tests for FSH, estradiol, and inhibin B can predict the chance of POI reversal [2]. Therefore, we call attention to the reversibility and irreversibility of animal POF models. For example, in both the genetic model and the oophorectomy model, it is not possible to reverse POF, while in models induced by chemotherapy, radiotherapy, and natural aging, it is possible to reverse ovarian activity due to the presence of ovarian stem cells, including very small embryonic‐like stem cells or VSELs, which are resistant to chemotherapy and radiotherapy. [1] and have also been observed in the ovaries of postmenopausal women [2].

Animal model studies provide an easier and more effective way to understand the occurrence and development of human diseases, but different methods of creating POF animal models have their advantages and disadvantages. Advantages of the chemotherapy‐induced POF animal model include a simple operation, low cost, short cycle and low mortality. Bleeding and myelosuppression are also side effects of the CTX‐induced POF animal model.^140^ Previous research has shown that about 10% to 30% of POF disorders are caused by autoimmune mechanisms.^182^ However, the stability of animal models of autoimmunity‐ and mental stress‐induced POF is low. A GAL‐induced animal model can mimic the features of physiological aging in patients with POF, but the success rate is lower, and the cycle time is longer.^140^ Therefore, choosing the ideal animal model for studying drug intervention and disease mechanisms is still a big challenge, and appropriate models should be selected according to the study's main purpose.

The most common causes of POF are exposure to chemotherapy and radiation therapy and the most common model of POF is induced by chemotherapy drugs. Chemotherapy‐related ovarian failure (COF) refers to ovarian endocrine and fertility dysfunction after exposure to chemotherapy drugs. Besides directly affecting follicles and eggs, chemotherapy may cause ovarian toxicity by affecting the entire ovary. Also, alkylating agents, the most toxic chemotherapy drugs, acting in a dose‐dependent manner, cause direct destruction of eggs and follicular emptying and may cause fibrosis and damage to ovarian blood vessels.^183^ Cyclophosphamide is the most gonadotoxic drug, and is also used in the clinic as the first line of chemotherapy drugs. It has been more widely used that other methods to create animal models. The therapeutic and toxic effects of cyclophosphamide are related to its active metabolites, such as acrolein and phosphoramide mustard. These metabolites bind to DNA and lead to disruption of DNA synthesis and cell death,^184^ which has harmful toxic consequences for cells in organs such as the heart, liver, and kidney and has severe toxic effects on the ovary.^17^, ^185^ CTX is the first chemotherapy drug to be associated with amenorrhea/POF and ovarian dysfunction^16^ and is also considered one of the most important gonadotoxic agents.^19^ Cyclophosphamide accelerates the maturation process of ovarian follicles into mature follicles, reduces ovarian reserves, and ultimately leads to premature ovarian failure in young female patients.^20^, ^186^ There is clear evidence that CTX leads to an up‐regulation of apoptosis in the ovary, as evidenced by rapid induction of DNA breaks^20^ and altered expression levels of pro‐apoptotic and anti‐apoptotic genes.^25^

The classic animal model for studying POF is created using chemotherapy drugs. The most common animal model of POF is induced by cyclophosphamide, and its toxic effect on the ovaries has been confirmed.^187^ Previous studies have shown that to create the most effective POF model, cyclophosphamide (120 mg/kg) must be used for at least 2 weeks and cisplatin (2 mg/kg) for more than ten days is recommended.^89^ In the study of Bahrebar et al., different concentrations of two chemotherapy drugs, cyclophosphamide, and busulfan, were investigated in order to create a POF model, and the use of 100 mg/kg CTX alone for ten consecutive days was chosen as the most effective POF induction model.^90^ In a meta‐analysis article, it has been shown that a 200 mg/kg dose with an 8 mg/kg maintenance dose of CTX for 14 consecutive days has the best efficacy in creating a CTX‐induced POF model. Cyclophosphamide has been used in many studies as a gonadotoxic chemotherapy drug to induce the POF model in mice and rats, as summarized in Table 2. Therefore, we suggest using cyclophosphamide to create an effective murine POF model. However, it is important to note that the choice of drugs may depend on the specific research objectives. On the other hand, the reported rates of premature ovarian failure models using chemotherapy drugs are very different and mainly rely on the chemotherapy protocols. The treatment depends on the number of injections, the age range, and the breed of the animal.

**TABLE 2:** Murine POF model induced by chemotherapy drugs.

Row	Species	Chemotherapy drug (dose)	Reference
1	ICR mice	Cyclophosphamide (75 mg/kg)	[38]
2	C57BL/6 mice	Cyclophosphamide (75 mg/kg)	[43]
3	C57BL/6 male × Balb/c female mice	Cyclophosphamide (75 mg/kg)	[36]
4	Balb/c mice	Cyclophosphamide (75 mg/kg)	[39]
5	C57BL/6 mice	Cyclophosphamide (100 mg/kg)	[40]
6	SD rats	Cyclophosphamide (100 mg/kg)	[23]
7	CD‐1 mice	Cyclophosphamide (100 mg/kg)	[54]
8	Kunming white mice	Cyclophosphamide (120 mg/kg)	[188]
9	Albino rats	Cyclophosphamide (150 mg/kg)	[10]
10	C57BL/6 mice	Cyclophosphamide (150 mg/kg)	[44]
11	Mice	Cyclophosphamide (150 mg/kg)	[42]
12	Wister albino rats	Cyclophosphamide (150 mg/kg)	[49]
13	Wister albino rats	Cyclophosphamide (150 mg/kg)	[51]
14	Wister albino rats	Cyclophosphamide (150 mg/kg)	[50]
15	B6D2F1 mice	Cyclophosphamide (50 mg/kg/14 days)	[53]
16	SD rats	Cyclophosphamide (50 mg/kg/1 day 8 mg/kg/14 days)	[48]
17	Wister albino rats	Cyclophosphamide (50 mg/kg/1 day 8 mg/kg/14 days)	[41]
18	Wister albino rats	Cyclophosphamide (50 mg/kg/1 day 8 mg/kg/14 days)	[22]
19	Albino rats	Cyclophosphamide (200 mg/kg/1 day 8 mg/kg/14 days)	[52]
20	Albino rats	Cyclophosphamide (200 mg/kg/1 day 8 mg/kg/14 days)	[4]
21	Albino rats	Cyclophosphamide (200 mg/kg/1 day 8 mg/kg/14 days)	[47]
22	BALB/c mice	Cyclophosphamide (75 100 150 mg/kg)	[45]
23	CD‐1 mice	Cyclophosphamide (75, 150 250 mg/kg)	[25]
24	NMRI mice	Cyclophosphamide (200 mg/kg/3 days)	[21]
25	C57BL/6 mice	Cyclophosphamide (300 mg/kg)	[26]
26	C57BL mice	Cyclophosphamide (1.2 mg/kg/14 days)	[37]
27	SPF mice	Cyclophosphamide (120 mg/kg) + Busulfan (30 mg/kg)	[85]
28	C57BL/6 mice	Cyclophosphamide (120 mg/kg) + Busulfan (30 mg/kg)	[87]
29	C57BL/6 mice	Cyclophosphamide (120 mg/kg) + Busulfan (30 mg/kg)	[88]
30	CD‐1 mice	Cyclophosphamide (120 mg/kg) + Busulfan (12 mg/kg)	[86]
31	ICR mice	Cyclophosphamide (120 mg/kg) + Busulfan (12 mg/kg)	[187]
32	C57BL/6 mice	Cyclophosphamide (120 mg/kg) + Busulfan (12 mg/kg)	[89]
33	Rat	Cisplatin (5 mg/kg)	[68]
34	Swiss mice	Cisplatin (5 mg/kg)	[64]
35	Wister albino rats	Cisplatin (5 mg/kg)	[71]
36	CD‐1 mice	Cisplatin (5 mg/kg)	[61]
37	Wister albino rats	Cisplatin (1.5 mg/kg/10 days)	[63]
38	CD‐1 (ICR) mice	Cisplatin (2.5 mg/kg)	[69]
39	ICR mice	Cisplatin (2 mg/kg/15 days)	[67]
40	C57BL/6 mice	Cisplatin (2 mg/kg/10 days)	[89]
41	C57BL/6 mice	Cisplatin (2 mg/kg/21 days)	[189]
42	CD‐1 mice	Cisplatin (2 mg/kg/15 days 5 mg/kg/8 days)	[70]
43	ICR mice	Doxorubicin (7.5 mg/kg)	[83]
44	CD‐1 mice	Doxorubicin (20 mg/kg)	[79]

Abbreviation: POF, premature ovarian failure.

## 4 CONCLUSION

In conclusion, animal POF models can help us understand the molecular mechanisms involved in the human disease better. According to the studies outlined, the most practical method to create an animal model of premature ovarian failure is to use cyclophosphamide because it causes the least damage to the animal and the death rate is lower. Although animal models of POF using chemotherapy drugs do not provide a complete picture of this disease, they are still an effective method of studying the disease and preventing the damage to ovarian function associated with chemotherapy. This review may help future researchers establish the most effective animal model of POF to develop therapeutic approaches and study the function and mechanism of POF. However, more studies are needed to choose the best POF model in different species and to find the most effective dose and frequency of injection.

## AUTHOR CONTRIBUTIONS

N.P. collected previous literature and wrote the manuscript. M.A. and H.Z. read and agreed to the final manuscript. R.F. reviewed and revised the manuscript. S.T. conceived the purpose and concept of the study, revised and finalized the manuscript. All authors read and approved the final manuscript.

## FUNDING INFORMATION

This research did not receive any specific grant from funding agencies in the public, commercial or not‐for‐profit sectors.

## CONFLICT OF INTEREST STATEMENT

The authors declare no competing interests.

## ETHICS STATEMENT

None.

## Data Availability

No data was used for the research described in the article.
